# Association between empathy and assertiveness of undergraduate students of medical colleges of Punjab

**DOI:** 10.3389/fpsyt.2025.1543308

**Published:** 2025-07-30

**Authors:** Fozia Fatima, Fuad Ahmad Siddiqui, Sabir Ali, Safia Fatima, Asiya Zahoor, Alina Alvi

**Affiliations:** ^1^ Department of Health Professions Education, National University of Medical Sciences, Islamabad, Pakistan; ^2^ Educational Development, University of Baltistan, Skardu, Pakistan; ^3^ Department of Pathology, PNS Shifa, Islamabad, Pakistan

**Keywords:** empathy, assertiveness, medical students, association, cross-sectional analysis

## Abstract

The study explores the relationship between empathy and assertiveness among undergraduate medical students in Punjab, Pakistan. Empathy, critical for patient care, enhances understanding and communication, while assertiveness is vital for advocacy and effective decision-making. Conducted as a cross-sectional analysis with 104 students from two medical colleges, the study used the Jefferson Scale of Physician Empathy (JSPE) and the Rathus Assertiveness Schedule (RAS) to evaluate these traits. Results reveal moderate levels of empathy and assertiveness, characterized by the recognition of emotional understanding’s importance in patient care, though practical application remains inconsistent. Assertiveness is higher in professional contexts but lower in personal boundary-setting, indicating gaps in interpersonal confidence. The study identifies no significant differences in empathy across gender, academic year, age, or college but finds that female students exhibit significantly higher assertiveness. Regression analysis shows a weak association between empathy and assertiveness, suggesting these traits develop independently and require distinct training. The findings highlight the need for structured educational interventions to bridge these gaps, such as narrative medicine, role-playing, and assertiveness workshops. Addressing cultural and academic challenges, the study underscores the importance of fostering empathetic and assertive behaviors in medical students to enhance both patient care and professional communication. This research contributes to optimizing medical education and preparing students for the complex demands of the healthcare profession.

## 1 Introduction

Empathy and assertiveness are two critical interpersonal competencies that significantly contribute to effective medical practice and high-quality patient care. In medical settings, empathy enables healthcare professionals to understand and resonate with the emotional experiences of patients, thereby fostering a therapeutic alliance that promotes trust, enhances communication, and improves adherence to treatment regimens (1). On the other hand, assertiveness equips medical professionals with the capacity to express their thoughts, needs, and decisions confidently and respectfully, ensuring effective advocacy for patients and timely clinical judgment (2). As noted by Zhuravlova et al. (3), the development of these interpersonal skills is essential for shaping competent, patient-centered physicians who can balance compassion with professional assertiveness.

In the context of Pakistan, especially in Punjab, the interplay between empathy and assertiveness is increasingly gaining attention due to the unique socio-cultural and systemic challenges present in the healthcare environment. Medical education institutions in Punjab have acknowledged the significance of empathy in enhancing the quality of healthcare delivery and have undertaken initiatives to incorporate structured empathy training within the curriculum. These efforts include the integration of simulated patient interactions, reflective practice exercises, and narrative medicine, all aimed at fostering emotional intelligence and patient-centered communication among students (4). Despite these initiatives, several barriers undermine the efficacy of empathy training. Academic competitiveness, and the emotionally taxing nature of medical education often lead to a decline in empathy as students’ progress through their training. Research by Fatima et al. (5) highlights the phenomenon of emotional desensitization among medical students, attributing it to prolonged clinical exposure, burnout, and insufficient institutional support. Furthermore, the socio-economic heterogeneity of Punjab’s patient population necessitates culturally sensitive care practices, making the development of empathy not only desirable but essential (6). However, inconsistencies in institutional resources and pedagogical approaches contribute to variability in how empathy is cultivated across different medical colleges. Claudel and Garcia Gonzalez (7) advocate for systematic interventions that address the root causes of empathy erosion, such as curricular overload, lack of psychosocial support, and inadequate emphasis on emotional well-being.

Assertiveness, though equally vital, has received comparatively less structured attention in the medical education curriculum in Punjab. Defined as the ability to express oneself confidently and maintain respectful communication without violating the rights of others, assertiveness is integral to managing interpersonal relationships, reducing stress, and maintaining professional boundaries (8). In educational settings, assertiveness empowers students to actively engage in academic discussions, seek clarifications, and contribute meaningfully to clinical dialogues (9). As observed by Grilo et al. (10), the traditional structure of medical education often reinforces passive learning and discourages questioning of authority, thereby impeding the development of assertive communication skills. The issue is further compounded by gender-based disparities, wherein female students face additional societal and institutional barriers that inhibit assertive expression. Guven and Unsal (11) argue that prevailing gender norms within South Asian societies often penalize assertiveness in women, perceiving it as non-conformist behavior, which in turn affects their confidence and participation in clinical settings. Hernandez-Umet et al. (12) emphasize the importance of institutional role models and inclusive learning environments in promoting assertiveness among students. Faculty members who model assertive behavior and encourage respectful dialogue can significantly influence students’ communication styles and professional identity formation. Conversely, excessive empathy without assertive boundaries can impair decision-making and lead to emotional exhaustion or professional inefficacy (13).

Therefore, a balanced integration of these competencies is necessary to produce well-rounded physicians who can communicate with compassion while maintaining professional confidence and decisiveness. Nikolaiev et al. (14) highlight the importance of designing curricula that recognize the dynamic interplay between these traits, allowing for the development of both emotional attunement and communicative clarity. Despite the critical role of these skills in medical practice, empirical research examining their interrelationship among medical students in Pakistan remains limited. Existing literature predominantly explores empathy and assertiveness as independent constructs, with insufficient attention paid to how they may influence each other within the clinical education environment. Patino-Dominguez (15) notes the need for localized studies that investigate these interpersonal competencies within specific cultural and institutional contexts. Understanding how empathy and assertiveness co-evolve among Pakistani medical students can inform evidence-based strategies to enhance interpersonal skill development in a manner that aligns with regional healthcare needs.

This study hypothesizes a significant positive correlation between empathy and assertiveness, suggesting that medical students who exhibit higher levels of one trait may be more likely to develop the other. The rationale behind this hypothesis lies in the premise that emotionally intelligent individuals possess both the sensitivity to understand others and the confidence to articulate their perspectives constructively (16). A balanced cultivation of these traits may enhance students’ overall communication skills, professional self-efficacy, and capacity for patient-centered care. Moreover, by exploring gender-based variations in the development of empathy and assertiveness, the study aims to identify potential disparities that could inform targeted interventions to promote equity in medical training (17). In conclusion, the concurrent development of empathy and assertiveness in medical students is essential for producing healthcare professionals who can deliver high-quality, patient-centered care in complex clinical environments. In Punjab, where cultural norms and systemic challenges shape medical education, there is a pressing need for comprehensive strategies that support the holistic development of these traits (18). By investigating the interrelationship between empathy and assertiveness, and accounting for gender-based differences, this study seeks to contribute to the enhancement of medical education practices. The findings are expected to inform the creation of inclusive, context-sensitive curricula that equip future physicians with the interpersonal tools necessary for effective practice in both local and global healthcare landscapes.

### 1.2 Objectives

1. To measure the levels of empathy and assertiveness in undergraduate medical students across different years of NUMS-affiliated medical colleges in Punjab.
2. To investigate an association between the level of empathy and assertiveness in undergraduate medical students across different years of NUMS-affiliated medical colleges in Punjab.

## 2 Theoretical framework

Empathy and assertiveness are critical interpersonal competencies in medical education, directly influencing patient care, professional communication, and decision-making (19). This study utilizes the Jefferson Scale of Physician Empathy (JSPE) and the Rathus Assertiveness Schedule (RAS) as its theoretical foundation, as both models provide operational definitions for these constructs and guide their assessment. The JSPE conceptualizes empathy as a fundamental skill in healthcare, comprising three dimensions: perspective-taking, compassionate care, and the ability to understand patients’ emotions (20). It emphasizes that empathy is both a cognitive and affective process essential for effective patient interactions. Research indicates that higher empathy levels in medical students lead to improved communication, better patient outcomes, and stronger doctor-patient relationships (21, 22). The JSPE serves as a validated tool for measuring these attributes, ensuring a reliable assessment of empathy in medical students (23). It developed at Thomas Jefferson University, the JSPE was introduced to address the need for a reliable tool to assess empathy in healthcare professionals (24). However, its reliance on self-reported responses introduces the risk of social desirability bias, where respondents may overestimate their empathy levels (25). Additionally, cultural variations in the expression of empathy may limit its universal applicability, as behaviors considered empathetic differ across regions (3, 26). While the JSPE remains an essential tool in medical education, it is often recommended to be used alongside other measures to enhance the accuracy of empathy assessment.

The RAS, on the other hand, defines assertiveness as the ability to express one’s thoughts, feelings, and needs confidently and respectfully (27). It is particularly relevant in medical education, where students must navigate complex professional hierarchies, engage in clinical decision-making, and communicate effectively with peers, faculty, and patients (28). Furthermore, it has demonstrated a strong correlation with related traits such as self-esteem and social confidence, reinforcing its relevance in evaluating interpersonal skills (29). However, the RAS has been criticized for its Western-centric approach, which may not fully account for cultural differences in communication styles (2). In cultures where indirect communication is valued, the scale may fail to recognize assertive behaviors that align with cultural norms. Additionally, the self-reported nature of the RAS can lead to biases, including underreporting due to self-doubt or overreporting influenced by perceived expectations (6). Despite these limitations, the RAS remains a valuable tool when used in conjunction with qualitative assessments or adapted to specific cultural contexts.

The Jefferson Scale of Physician Empathy (JSPE) and the Rathus Assertiveness Schedule (RAS) provide the theoretical foundation for this study by operationally defining empathy and assertiveness. The JSPE conceptualizes empathy as a multidimensional construct in medical practice, encompassing cognitive, affective, and behavioral components that influence patient-centered care. It provides a structured approach to assessing how medical students understand and respond to patients’ emotions, reinforcing the importance of empathy in clinical decision-making. Similarly, the RAS defines assertiveness as a key interpersonal skill involving confident self-expression, effective communication, and the ability to advocate for oneself and others. The use of RAS allows for a systematic evaluation of assertive behaviors, particularly in hierarchical and professional settings. By integrating these validated models, this study examines the interplay between empathy and assertiveness within the medical education context, ensuring a theoretically grounded exploration of these traits.

This study seeks to address a gap in the literature by examining the association between empathy and assertiveness among undergraduate medical students in Punjab, Pakistan, with a particular focus on gender-related differences. Empathy is essential in fostering trust, improving patient adherence to treatment, and enhancing the overall quality of care, while assertiveness facilitates effective communication, decision-making, and patient advocacy (30, 31). Despite the recognized importance of these attributes in medical practice, limited research has explored their simultaneous development within the context of medical education, particularly in a culturally diverse and resource-constrained setting such as Punjab. Medical students in Punjab face distinct challenges that may impact the cultivation of these skills, including high academic pressure, hierarchical institutional structures, and sociocultural expectations that can hinder assertiveness, particularly among female students (11). While medical curricula have increasingly incorporated empathy training through simulated patient interactions, reflective practice, and narrative medicine, disparities in institutional resources and pedagogical approaches contribute to inconsistencies in its implementation and long-term retention (7). Additionally, prolonged clinical exposure and emotional desensitization have been linked to empathy erosion, necessitating systematic interventions such as mentorship programs, stress management workshops, and structured experiential learning opportunities (5). Conversely, assertiveness remains an underemphasized skill in medical training, often overshadowed by cultural norms that prioritize deference to authority, thereby limiting students’ confidence in expressing their opinions and advocating for patients (10). Therefore, in this study, empathy acts as the independent variable because it is posited to influence the outcome (assertiveness). Assertiveness is the dependent variable because it is hypothesized to change depending on the level of empathy in medical students.

*Hypothesis: There is no significant association between the level of empathy and assertiveness in undergraduate medical students across different years of NUMS-affiliated medical colleges in Punjab.*

## 3 Materials and methods

### 3.1 Study design

This study was a cross-sectional analytical design to investigate the association between empathy and assertiveness among undergraduate medical students in Pakistan. The principal investigator had taken ethical permission from the IRB and Ethical Committee of the National University of Medical Sciences, Pakistan (Ref No: 06/IRB&EC/NUMS/34).

### 3.2 Study population

The population of this study was the undergraduate students of the medical colleges of Punjab. Due to time and resource constraints, the students of two medical colleges were used as the targeted population in the current study. From these two settings, researchers only targeted 4th and 5th years students. Fourth and fifth-year medical students were chosen for this study because they have significant clinical exposure, allowing them to apply empathy and assertiveness in real patient interactions. Their advanced stage of training provides a more accurate assessment of these interpersonal skills in professional settings. Additionally, selecting students from the final years ensures that their experiences reflect the impact of medical education on the development of these competencies.

The number of undergraduate students from the 4th and 5th years in these two medical colleges was 140. While the sampling fraction (74.3%) is high, the approach remains within the scope of simple random sampling (SRS), as every student had an equal probability of being selected, and the required sample size was determined through a well-established formula. However, this study explicitly stated the method of randomization (e.g., using a random number generator or lottery system) to further validate the claim of SRS and avoid ambiguity.

### 3.3 Sample and sampling technique

The total number of accessible populations was 140 in the two medical colleges (See **Table 1**). The researchers utilized the Yamane Formula for calculating the sample size and it was also confirmed by Morgan and Krejcie table for determining the sample from the targeted population.


$$Yamane\;Formula\;n=\frac{N}{1+N\;\left(e\right)2}$$

**Table 1:** Accessible population of medical colleges.

NO	Medical colleges	Undergraduate students
1	Fazaia Medical College	70
2	HITEC Institute of Medical Sciences, Taxila	70
Total		140

Where n = sample size,

N = total population (Number of Students of 2 NUMS-affiliated medical colleges).

E = margin of error.


$$n=\frac{140}{1\;\pm \;140\;\left(0.05\right)2}=103.7=104$$

The researchers took 104 undergraduate students, both male and female from two NUMS-affiliated medical colleges in Punjab as the sample of this study by using a simple random sampling technique. The study included undergraduate medical students currently enrolled in Fazaia and HITEC medical colleges, specifically selecting those in their fourth and fifth years of study. This criterion was established to ensure a comprehensive analysis of students in the advanced stages of their medical education, where clinical exposure and professional development are more pronounced. Exclusion criteria were applied to maintain the integrity and relevance of the study. Students who had completed their undergraduate medical education and were engaged in postgraduate studies or other advanced medical training were excluded, as their experiences and perspectives may differ significantly from those still in undergraduate training. Additionally, participants who did not complete the entire survey or provided incomplete responses to the Jefferson Scale of Physician Empathy (JSPE) and the Rater Agreement Scale (RAS) questionnaires were excluded to ensure data reliability and consistency in analysis.

Jefferson Scale of Physician Empathy (JSPE), a validated self-report questionnaire was used that was specifically designed to measure empathy in medical students and professionals (20). The JSPE consists of 19 items scored on a 5-point Likert scale, enabling participants to self-report their attitudes and behaviors related to empathy. These items assess dimensions such as perspective-taking, compassionate care, and understanding patient emotions. The tool has been widely adopted in medical education research to evaluate interventions designed to enhance empathy and to track changes in empathy levels over time. Its psychometric properties have been extensively validated across diverse populations, making it a gold standard in empathy measurement. Over the years, the JSPE has contributed significantly to understanding the role of empathy in healthcare and shaping curricula aimed at fostering this critical skill in medical professionals.

Rathus Assertiveness Schedule (RAS) is a widely used self-report inventory to measure assertiveness (Harris & Brown, 1979). The RAS consists of 25 statements, each rated on a 5-point Likert scale, which captures various aspects of assertive behavior, including the ability to express thoughts, feelings, and needs confidently and respectfully. Its development was influenced by the growing recognition of assertiveness as a balance between passivity and aggression, making it a cornerstone in behavioral therapies and educational programs. Over the decades, the RAS has been utilized extensively in diverse settings, including medical education, to evaluate students’ communication skills, confidence levels, and ability to handle interpersonal challenges. Its robust psychometric validation has cemented its reputation as a reliable and effective tool for measuring assertiveness. Data collection was conducted using online surveys distributed through institutional email lists.

### 3.5 Validity and reliability of scales in Pakistani context

A panel of five experts in medical education, psychology, and behavioral sciences evaluated the questionnaire for content validity. The experts were selected based on their qualifications, including PhDs and postgraduate degrees, as well as their extensive experience in research and teaching. Content Validity Ratio (CVR) was calculated using Lawshe’s (1975) formula, with all five experts unanimously agreeing that each item was essential, resulting in a CVR of 1.0. This unanimity confirms that the questionnaire accurately measures the intended constructs. The CVR was determined as follows:


$$CVR=ne-\frac{\;N/2}{N/2}$$

Where e is the number of panel members who say an item is “essential,” n is the total number of panel members, and CVR is the content validity ratio. By using this formula


$$CVR=5-\frac{\;5/2}{5/2}$$

$$CVR=5-\frac{\;5/2}{5/2}=5-\frac{\left(2.5\right)}{\left(2.5\right)}=\frac{\left(2.5\right)}{\left(2.5\right)}=1$$

The assessment is well-represented and valid, achieving a CVR score of 1 for content validity, indicating the tool’s maximum degree of application.

**Table 2** shows that the Rathus Assertiveness Schedule (RAS) was selected for this study after undergoing pilot testing to ensure its validity and reliability in the Pakistani context. Content validity was confirmed using expert evaluation, yielding a Content Validity Ratio (CVR) of 1.0, indicating strong agreement on its relevance. Reliability analysis demonstrated high internal consistency, with a Cronbach’s alpha of 0.850 for assertiveness. Necessary cultural adaptations were made based on feedback to align the scale with local communication norms. Given its strong psychometric properties and proven suitability, the RAS was deemed an appropriate tool for assessing assertiveness in Pakistani medical students (See **Table 2**).

**Table 2:** Reliability of Scales.

Dimensions of scale	No of items	Cronbach alpha
A-Empathy of students	19	.764
a. Cognitive Ingredients of Empathy	10	.851
b. Compassionate Care	09	.724
B-Assertiveness of Students	25	.850

### 3.6 Data collection and data analysis

For this project, a structured Google Form was designed to collect data on empathy and assertiveness among undergraduate medical students from medical colleges in Punjab. The form included a series of validated questionnaire items measuring students’ empathy and assertiveness levels. After finalizing the form, email was chosen as the primary mode of distribution to ensure wide and efficient dissemination across all target colleges. Email invitations containing the Google Form link were sent to the official email addresses of participating students, along with clear instructions regarding confidentiality and consent. Students were informed that their responses would remain anonymous and would be used solely for research purposes. Follow-up reminders were sent periodically to ensure a high response rate. The data collection phase lasted for two weeks, during which a total of 104 responses were recorded.

The collected data was analyzed using a combination of descriptive and inferential statistical methods. Descriptive statistics, including mean, standard deviation, and frequency distributions, were calculated to summarize empathy and assertiveness levels. Inferential techniques, Regression analysis was performed to evaluate the relationship between predictors (Cognitive Ingredients of Empathy and Compassionate Care) and assertiveness. Tests of normality, including the Kolmogorov-Smirnov and Shapiro-Wilk tests, were conducted to ensure that the data met parametric assumptions. All statistical analyses were performed using statistical software, ensuring accurate and reliable results to support the research objectives.

In this study, careful measures were taken to ensure methodological rigor and transparency in sampling, data collection, and analysis. The selection of Fazaia Medical College and HITEC Institute of Medical Sciences was based on their affiliation with the National University of Medical Sciences (NUMS), ensuring that the study population represents medical students undergoing a standardized curriculum aligned with national medical education standards. Given time and resource constraints, these two colleges were chosen as they provide a reasonable and structured representation of the target population, ensuring meaningful generalizability of the findings. A simple random sampling technique was employed to minimize selection bias and ensure fair representation. To implement this method, a computer-generated random number sequence was used. Each eligible student was assigned a unique identifier, and participants were selected through an automated process, ensuring objectivity and replicability. This approach strengthens the validity of the findings and allows future researchers to replicate the methodology in similar academic settings.

The study acknowledges the inherent limitations of its cross-sectional design. Since data were collected at a single time point, the study does not establish causal relationships between empathy and assertiveness. However, to mitigate this limitation, a rigorous statistical approach was applied to examine the association between these traits comprehensively. While changes in empathy and assertiveness over time were beyond the study’s scope, future longitudinal research could further explore these variations and provide deeper insights into their development.

The data collection process incorporated both online and paper-based surveys to maximize participation and ensure accessibility. To maintain consistency between the two collection methods, identical formats were used, and responses were systematically reviewed. Measures were taken to verify data integrity, with incomplete responses excluded from the final analysis. The study maintained strict quality control procedures, ensuring uniformity in data entry and validation, thereby minimizing errors or discrepancies between different modes of survey administration. By incorporating these methodological safeguards, this study ensures a robust and reliable investigation of the association between empathy and assertiveness among medical students. These measures enhance the credibility and applicability of the findings, contributing to the development of effective educational strategies for fostering these critical interpersonal skills in future healthcare professionals.

## 4 Results

**A- Descriptive statistics**

**Figure 1:**
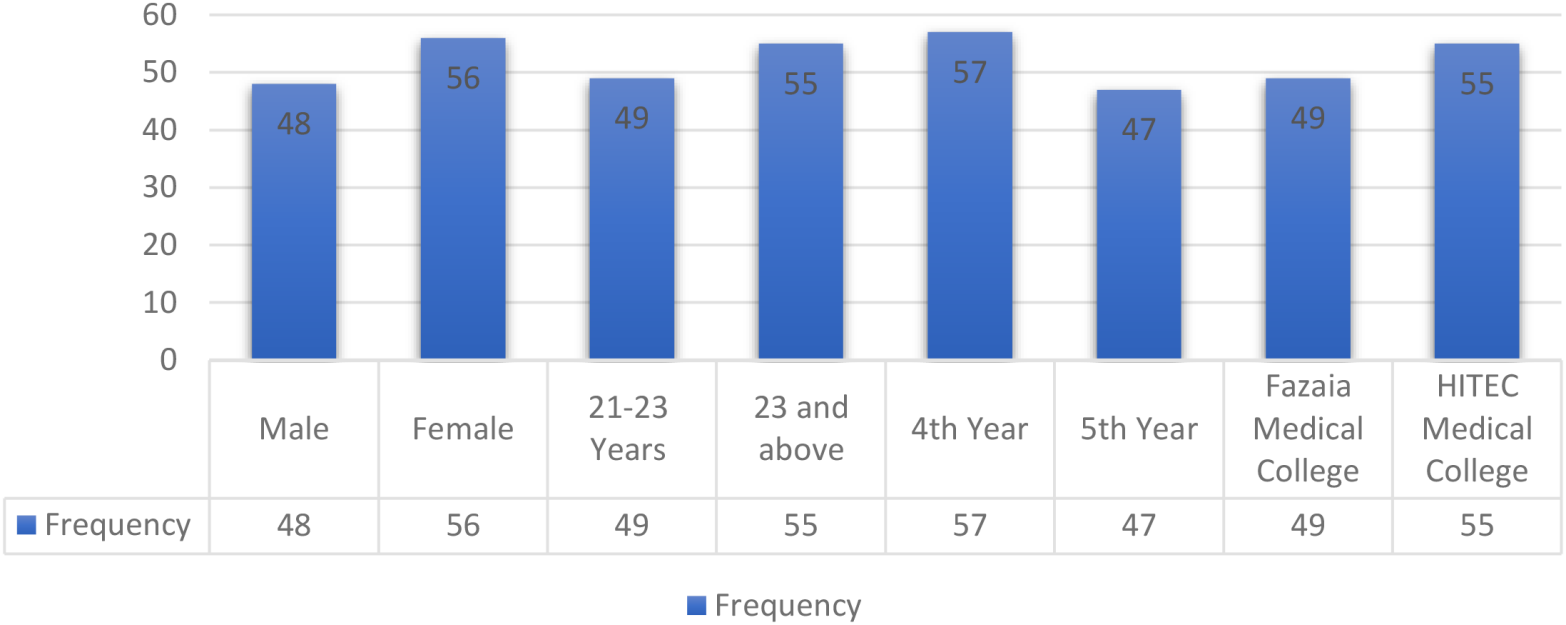
Demographic information.

In **Figure 1**, the bar chart illustrates the frequency and percentage distribution of various demographic and academic characteristics among participants. Females show a slightly higher representation (53.8%) compared to males (46.2%). Participants aged 23 and above comprise 52.9%, slightly higher than those aged 21–23 years (47.1%). Fourth-year students have the highest representation at 54.8%, while fifth-year students are the least represented at 45.2%. Among institutions, HITEC Medical College has a higher representation (52.9%) compared to Fazaia Medical College (47.1%). This data reflects a balanced demographic and academic spread, with notable variations across certain categories.

**Table 3** shows the data on students’ responses towards empathy reveal an overall mean score of 3.127, indicating a moderate empathetic attitude among medical students. The highest agreement was observed in items emphasizing the importance of understanding patients’ emotions in care, such as “My patients feel better when I understand their feelings” (mean = 4.51) and “Empathy is a therapeutic skill without which treatment success is limited” (mean = 4.23). However, a lower agreement was noted for statements that dismiss the role of emotions in medical treatment, such as “I believe that emotion has no place in the treatment of medical illness” (mean = 2.26). This suggests that while students recognize the importance of empathy, some variability exists in their ability to consistently apply it in practice. Additionally, moderate responses to recognizing non-verbal cues and imagining oneself in a patient’s perspective highlight areas for development.

**Table 3:** Students’ responses about their empathy and assertiveness (N=104).

1- Student’s responses towards empathy	Mean	Std. Deviation
1. My understanding of how my patients and their families feel does not influence medical or surgical treatment	2.83	1.260
2. My patients feel better when I understand their feelings	4.51	.596
3. It is difficult for me to view things from my patient’s perspectives	2.46	1.027
4. I consider understanding my patient’s body language as important as verbal communication in caregiver-patient relationships.	4.28	.847
5. I have a good sense of humor that I think contributes to better clinical outcome	3.83	.908
6. Because people are different it is difficult for me to see things from my patient’s perspectives	2.73	1.031
7. I try not to pay attention to my patients’ emotions in history taking or in asking about their physical health	2.58	1.121
8. Attentiveness to my patient’s personal experience does not influence treatment outcomes	2.63	1.078
9. I try to imagine myself in my patient’s shoes when providing care to them	3.63	.830
10. Patient’s illnesses can be cured only by medical or surgical treatment, therefore, emotional ties to my patients do not have a signification flounce on medical or surgical outcomes	2.46	1.123
11. Asking patients about what is happening in their personal lives does not help understand their physical complaints	2.39	1.061
12. I try to understand what is going on in my patient’s minds by attention to their non-verbal cues and body language	3.92	.893
13. I believe that emotion has no place in the treatment of medical illness	2.26	1.068
14. Empathy is a therapeutic skill without which success in treatment is limited	4.23	.743
15. An important component of the relationship with my patients is my understanding of their emotional status, as well as that of their families	3.93	.803
16. I try to think like my patients to render better care	3.90	.806
17. I do not allow myself to be influenced by strong personal bonds between my patients and their family members	3.15	1.049
18. I do not enjoy reading non-medical literature or the arts	2.53	1.159
19. I believe that empathy is an important therapeutic factor in medical or surgical	4.29	.773
**Students’ Empathetic Attitude**	**Mean=3.127**	
2- Students’ response to their assertiveness		
1. Refusing a request made by a person in authority	3.27	1.011
2. Telling someone that you think he/she treated you unfairly	3.45	1.056
3. Asking a person to stop doing something that annoys you	3.46	.954
4. Refusing unsatisfactory goods or services	3.68	.857
5. Discussing with someone your impression that they are trying to avoid you	3.46	.839
6. Refusing to lend something to a near acquaintance	3.05	1.019
7. Insisting that someone does his/her share in a joint task	3.44	1.006
8. Asking someone to explain something you have not understood	3.78	.891
9. Telling someone who has justly criticized you that he/she is right	3.45	.975
10. Asking someone whether you have hurt him/her	3.53	1.017
11. Saying that you are sorry when you have made a mistake	3.79	.977
12. Asking someone to show you the way	3.60	1.013
13. Admitting that you know little about a particular subject	3.71	.963
14. Starting a conversation with a stranger	3.45	.875
15. Telling a group of people about something you have experienced	3.50	.900
16. Joining in the conversation of a group of people	3.33	.972
17. Maintaining your own opinion against a person who has a very pronounced opinion	3.55	.975
18. Giving your opinion to a person in authority	3.52	.899
19. Going up to someone to make their acquaintance	3.38	.914
20. Acknowledging a compliment about your appearance	3.68	.857
21. Telling someone that you like him/her	3.37	.866
22. Telling someone that you are fond of him/her	3.33	.894
23. Acknowledging a compliment on something you have done	3.55	.839
24. Saying that you enjoy the experience of being told that you are liked	3.34	.994
25. Saying that you enjoy people telling you that they are very fond of you	3.26	.977
**Assertiveness of Medical Students**	**Mean= 3.19**	

For assertiveness, the data reflect an overall mean score of 3.19, indicating moderate levels of assertive behavior among medical students. Students demonstrated higher assertiveness in professional contexts, such as “Saying that you are sorry when you have made a mistake” (mean = 3.79) and “Asking someone to explain something you have not understood” (mean = 3.78). However, lower scores were seen in personal challenges, such as “Refusing to lend something to a near acquaintance” (mean = 3.05), suggesting areas where confidence and boundary-setting could be improved. These findings underscore the need for educational programs to foster a balance between empathy and assertiveness, equipping medical students with the interpersonal skills essential for patient-centered care and professional communication.

The analysis of demographic data revealed a balanced representation of participants across gender, age, academic year, and institution, ensuring a comprehensive understanding of medical students’ attitudes. Findings on empathy indicated a moderate overall empathetic attitude (mean = 3.127), with strong recognition of its therapeutic importance but some inconsistencies in applying it, particularly in interpreting non-verbal cues and adopting patients’ perspectives. Similarly, assertiveness levels were moderate (mean = 3.19), with higher confidence in professional communication, such as admitting mistakes and seeking clarification, but weaker boundary-setting in personal interactions. These results suggest that while medical students acknowledge the value of both empathy and assertiveness, they may struggle with their consistent application. Enhancing medical education through targeted training in emotional intelligence, non-verbal communication, and assertiveness can bridge these gaps, ensuring that future physicians develop both compassionate patient care skills and confident professional communication abilities.

**B-Inferential Statistics**

In **Table 4**, the Kolmogorov-Smirnov and Shapiro-Wilk tests were used to assess the normality of the data for Empathy and Assertiveness. While the K-S test suggests that empathy and assertiveness do not significantly deviate from normality (p = 0.071, p=0.078), the S-W test also indicates a not significant deviation (p = 0.071, p= 0.083). These results suggest that the data is normally distributed, warranting the use of parametric statistical methods for further analysis.

**Table 4:** Tests of normality.

Variables	Kolmogorov-Smirnov^a^			Shapiro-Wilk		
	Statistic	df	Sig.	Statistic	df	Sig.
Empathy	.086	98	.071	.929	98	.071
Assertiveness	.106	98	.078	.958	98	.083

a. Lilliefors Significance Correction.

The regression analysis (**Table 5**) indicates a weak relationship between the predictors, “Cognitive Ingredients of Empathy” and “Compassionate Care,” and the outcome variable, as shown by the low R-value (.196) and R Square (.038). This means only 3.8% of the variance in the outcome is explained by these predictors, with limited predictive power and a standard error of 10.61 indicating variability in predictions.

**Table 5:** Regression analysis for association between empathy and assertiveness of medical students.

Model	R	R square	Adjusted R square	Std. error of the estimate
1	.196^a^	.038	.018	10.60946

a. Predictors: (Constant-Empathy), Cognitive Ingredients of Empathy, Compassionate Care.

The regression analysis (**Table 6**) shows that the predictors, “Cognitive Ingredients of Empathy” and “Compassionate Care,” do not significantly explain variations in assertiveness (F(2,95)=1.896,p=.156F(2, 95) = 1.896, p = .156F(2,95)=1.896,p=.156). While the total variance is 11,120.122, the regression model accounts for only 426.867, with the majority attributed to residuals. This indicates that the predictors have limited explanatory power for assertiveness.

**Table 6:** ANOVA.

Model		Sum of squares	df	Mean square	F	Sig.
1	Regression	426.867	2	213.434	1.896	.156^b^
	Residual	10693.255	95	112.561		
	Total	11120.122	97			

a. Dependent Variable: Assertiveness.

b. Predictors: (Constant), Cognitive Ingredients of Empathy, Compassionate Care.

The regression analysis (**Table 7**) indicates that neither “Cognitive Ingredients of Empathy” (TCIOE; B=−0.079,p=.760B = -0.079, p = .760B=−0.079,p=.760) nor “Compassionate Care” (CCOE; B=0.581,p=.062B = 0.581, p = .062B=0.581,p=.062) significantly predicts assertiveness. While the constant (B=66.599,p<.001B = 66.599, p <.001B=66.599,p<.001) is significant, the predictors show minimal influence, with “Compassionate Care” approaching significance but not reaching it.

**Table 7:** Coefficient.

Model		Unstandardized coefficients		Standardized coefficients	t	Sig.
		B	Std. Error	Beta		
1	(Constant)	66.599	10.463		6.365	.000
	CIOE	-.079	.257	-.034	-.307	.760
	CCOE	.581	.308	.207	1.888	.062

a. Dependent Variable: Assertiveness.

*CIOE, Cognitive Ingredients of Empathy.

*CCOE, Compassionate Care OF Empathy.

## 5 Discussion

The discussion section of this study provides critical insights into the complex relationship between empathy and assertiveness among undergraduate medical students, emphasizing the need for a nuanced understanding of these interpersonal skills. The findings indicate that while students exhibit a moderate level of empathy (mean = 3.127), there are inconsistencies in its practical application, reflecting the challenges of translating theoretical understanding into clinical practice. High agreement with statements emphasizing empathy’s role in patient care suggests that students acknowledge its significance, aligning with previous research that underscores the importance of emotional intelligence in medical professions (1, 4). Emotional intelligence plays a crucial role in medical education, as it enables students to build meaningful connections with patients, leading to improved treatment adherence and overall satisfaction (23). However, lower agreement with statements related to non-verbal communication and perspective-taking suggests potential deficiencies in active empathetic engagement, reinforcing the concerns raised by previous studies regarding empathy erosion during medical training due to emotional desensitization and high clinical workload (7). This phenomenon has been widely reported in medical education literature, with studies demonstrating a decline in empathy as students’ progress through their training, attributed to increased exposure to emotionally taxing situations and a focus on biomedical aspects of care at the expense of humanistic skills (5). To counteract this decline, structured interventions such as role-playing, simulated patient encounters, and reflective narrative medicine exercises have been recommended, providing students with opportunities to practice empathy in a controlled yet realistic environment (11).

Assertiveness, with an overall mean of 3.19, exhibited a stronger presence in professional settings, particularly in tasks such as admitting mistakes and seeking clarifications, but was weaker in personal interactions involving boundary-setting. This aligns with findings that highlight the importance of assertiveness in medical education, where students are expected to communicate effectively, advocate for their patients, and assert their professional opinions confidently (22). Assertiveness is a critical skill for physicians, as it enables them to navigate complex clinical environments, interact with colleagues across hierarchical structures, and maintain ethical decision-making under pressure (20). The significant gender difference in assertiveness, with female students scoring higher than their male counterparts, supports previous research indicating that women in medical professions often adopt more assertive communication styles to navigate professional hierarchies and counter gender biases (24, 25). This trend may reflect the evolving role of women in medicine, as increasing numbers of female physicians are challenging traditional stereotypes and advocating for their professional autonomy (12). However, cultural and societal expectations may still shape assertive behavior differently across genders, as male students might experience pressure to conform to traditional models of deference within hierarchical medical structures, leading to lower assertiveness scores in personal interactions (10). The lack of significant differences in assertiveness across academic years, age groups, and institutional affiliations suggests a relatively uniform training environment, yet it also indicates a need for targeted assertiveness training programs that address specific challenges faced by students at different stages of their education.

The regression analysis revealed a weak relationship between empathy and assertiveness, explaining only 3.8% of the variance, suggesting that these attributes develop independently rather than as interdependent traits. This finding aligns with previous research that argues empathy and assertiveness require distinct developmental pathways, as they serve different functions in medical communication (13). While empathy fosters a patient-centered approach that prioritizes understanding and emotional connection, assertiveness is essential for decision-making, advocacy, and conflict resolution, making it necessary to cultivate both skills without compromising one for the other (14). This distinction highlights the importance of implementing separate but complementary training programs, ensuring that students do not struggle to balance excessive emotional involvement with the need for firm and confident communication. Previous studies have emphasized that medical curricula must incorporate structured training that not only fosters empathy through humanistic and patient-centered approaches but also strengthens assertiveness through practical exercises such as negotiation skills workshops, role-playing, and scenario-based learning (15). Furthermore, cross-cultural studies have demonstrated that assertiveness training should be adapted to local healthcare contexts, acknowledging the impact of hierarchical medical structures and sociocultural expectations on students’ ability to express themselves confidently (3). The integration of such training programs can help ensure that future physicians possess the interpersonal competencies necessary to deliver compassionate yet decisive patient care.

The study’s results directly address its three research objectives, providing a strong rationale for the incorporation of targeted training interventions in medical education. The first objective, measuring empathy and assertiveness levels among medical students, was effectively achieved by identifying moderate levels of both traits, with notable gender differences in assertiveness. The second objective, investigating the association between these traits, was examined through regression analysis, revealing a weak link that supports the independent development of empathy and assertiveness. This finding further reinforces the argument that medical curricula should treat these attributes as distinct yet complementary skills requiring tailored instructional strategies (2). This suggests that existing medical training programs may provide a consistent foundation for assertiveness development but require additional efforts to ensure equitable growth of this skill across all student populations. Given the increasing emphasis on patient-centered care and interprofessional collaboration, these findings highlight the necessity for structured educational interventions that foster both empathetic engagement and assertive communication in future physicians (6). Addressing these competencies through a well-designed curriculum can ultimately enhance professional confidence, patient satisfaction, and overall healthcare quality, ensuring that medical graduates are well-equipped to navigate the challenges of modern clinical practice.

## 6 Conclusions

1. Students exhibit higher empathy in professional aspects, such as understanding patients’ emotions and recognizing the importance of empathy in treatment, but face challenges in consistently applying these skills across contexts. Similarly, assertiveness is stronger in professional scenarios, like seeking clarity or acknowledging mistakes, but weaker in personal interactions, such as setting boundaries. These moderate levels highlight the need for targeted skill enhancement.
2. The regression analysis indicates a weak relationship between empathy-related predictors and assertiveness, explaining only 3.8% of the variance. This implies that while both traits are important for medical professionals, they may develop independently and require separate training programs to enhance each skill effectively.

## 7 Recommendations

1. Medical educators and clinical instructors should organize structured empathy workshops using role-playing, simulated patient interactions, and reflective exercises to help students apply empathy consistently across clinical settings. Real-case scenarios should be integrated to bridge the gap between theoretical knowledge and practical application.
2. Medical college administrators, in collaboration with communication skills trainers, should implement simulation-based assertiveness training programs focusing on standardized patient interactions, conflict-resolution simulations, and case-based discussions to enhance professional and personal assertiveness.
3. Student development committees and faculty mentors should design gender-sensitive communication coaching sessions, encouraging female students to refine leadership and negotiation skills while providing male students with confidence-building exercises to strengthen assertive communication.
4. Curriculum committees and academic policymakers should develop separate but complementary skill development programs for empathy and assertiveness, ensuring empathy training emphasizes emotional intelligence and patient-centered care while assertiveness training focuses on decision-making, advocacy, and professional communication, with periodic evaluations to measure effectiveness.

## Data Availability

The raw data supporting the conclusions of this article will be made available by the authors, without undue reservation.
